# *Lycium barbarum* glycopeptide targets PER2 to inhibit lipogenesis in glioblastoma by downregulating SREBP1c

**DOI:** 10.1038/s41417-023-00611-4

**Published:** 2023-04-17

**Authors:** Jian Yao, Jian-wen Hui, Yan-jun Chen, Dong-yang Luo, Jiang-shu Yan, Yi-fan Zhang, Yuan-xiang Lan, Xiu-rui Yan, Zhi-hua Wang, Heng Fan, He-chun Xia

**Affiliations:** 1 Ningxia Medical University, Yinchuan, Ningxia Hui Autonomous Region 750004 P.R. China; 2 Department of Neurosurgery, General Hospital of Ningxia Medical University, Yinchuan, Ningxia Hui Autonomous Region 750004 P.R. China; 3 Ningxia Key Laboratory of Stem Cell and Regenerative Medicine, Institute of Medical Sciences, General Hospital of Ningxia Medical University, Yinchuan, Ningxia, Autonomous Region 750004 China

**Keywords:** Biomarkers, Cancer metabolism

## Abstract

*Lycium barbarum* polysaccharide (LBP) is a substance with various biological activities extracted from *Lycium barbarum*. LbGPs are peptidoglycans with a short peptide backbone and a complex, branched glycan moiety, which is further extracted and isolated from LBPs. Previous studies have shown that LbGP can inhibit cancer cell growth, but its specific mechanism is not completely clear. In this study, we found that LbGP could inhibit the proliferation of glioma cells and promote the expression of period 2 (PER2) through the PKA-CREB pathway. In addition, LbGP could inhibit the de novo synthesis of lipids by downregulating SREBP1c and its target genes, which depended on the expression of PER2. Moreover, PER2 negatively regulated the expression of SREBP1c via suppressing PI3K/AKT/mTOR pathway. In summary, LbGP may upregulate the expression of PER2 to reduce the expression of SREBP1c, inhibit lipid synthesis in glioblastoma, and inhibit glioblastoma cell proliferation. This study provides an alternative drug for the treatment of glioma and elucidates its potential mechanism.

## Introduction

In China, *Lycium barbarum*, as a traditional Chinese medicine, has been used for thousands of years. The effects of *L. barbarum* are attributed to the polysaccharides (LBPs) it contains, which have diverse activities, such as antioxidant effects, antiaging effects, anti-inflammatory effects, reproductive protection, liver protection, eye protection, and immune regulation [1]. In addition, LBP has been reported to inhibit the growth of hepatoma cells, colon cancer cells, gastric cancer cells and cervical cancer cells [2–5]. LbGP is a further extract of LBP, which is linked by glycoside oligopeptides to obtain five glycoconjugates, LbGP1-LbGP5, which are collectively called *Lycium barbarum* glycopeptides. There are currently few studies on the biology of LbGP.

Glioblastoma (GBM) originates from glial cells and is the most common intracranial malignant tumor, with an annual incidence of approximately 3–6.4/100,000. GBM is the most common primary malignant central nervous system tumor and shows high invasiveness and frequent recurrence. Glioma has always been an intractable disease in the field of neurosurgery. Although many clinical treatment strategies for gliomas, including surgery, chemotherapy, radiotherapy, and targeted therapy, have been studied and improved over the years, the prognosis of patients has not been improved [6]. The overall median survival time is approximately 14 months, and less than 10% of patients have a survival time of more than 2 years. Further elucidation of the mechanism of glioma occurrence and growth is needed to identify relatively specific targets and to improve the prognosis and curative effect of glioma patients [7–9].

Circadian genes are present in all nucleated cells, and these genes are involved in regulating the rhythm of all periodic processes, controlling cell metabolism, reproduction, differentiation, senescence, inflammation and many other function [10]. Circadian genes modulate the rhythmic transcription of numerous genes in all cells by a mechanism that relies on coordinated chromatin remodeling events [11]. Clock circadian regulator (CLOCK) and brain and muscle Arnt-like protein-1 (BMAL1) heterodimers are formed to rhythmically activate the expression of period 1/2/3 (Per1, Per2 and Per3) and cryptochrome 1/2 (Cry1 and Cry2), forming complex to inhibit CLOCK and BMAL1 transcriptional activities. Moreover, increasing evidence has shown that PER2 acts as a tumor suppressor gene to inhibit the proliferation of tumor cells [7]. In brief, the clock gene is closely related to substance metabolism, and PER2 plays a critical role in tumor growth and metabolism.

Sterol regulatory element binding protein (SREBP) is an important nuclear transcription factor that belongs to the family of basic helix-loop-helix zippers. SREBP1c is a subtype of SREBP that mainly promotes fatty acid synthesis. Its target genes (FASN, SCD, ACACA, etc.) regulate various specific processes of fatty acid synthesis [12]. Previous studies have shown that tumor cell proliferation, migration, invasion and apoptosis are directly related to lipid synthesis and metabolism [13]. Most of the components of the cell membrane are phospholipids, and cell proliferation depends on the synthesis of phospholipids [12]. The growth and proliferation of gliomas depend on the expression of SREBP1c [8]. SREBP1c and its downstream genes play a crucial role in glioma growth.

Despite major efforts to treat gliomas, no effective treatments are currently available. The link between the tumor biorhythm and metabolism is still unclear. The present study evaluated the effects of LbGP on glioma cell proliferation, migration, invasion and apoptosis in vivo and in vitro. We further discussed the connection between circadian genes and lipid metabolism. The present results revealed a novel mechanism of action of PER2, underlying the role of PER2 in lipid metabolism, as well as a new drug to prevent glioma progression.

## Methods

### Reagents and antibodies

Antibodies against phospho-CREB (Ser133) (87G3) and CREB (48H2) were purchased from Cell Signaling Technology; anti-SREBP1 (2A4) (sc-13551) and anti-PER2 (ab227727) for western blot (WB) was purchased from Santacruz Biotechnology; anti-mTOR antibody (ab32028), anti-p-mTOR (ab137133) and anti-PER2 (ab179813) for WB was purchased from Abcam; and anti-PER2 (ab227727) and anti-SREBP1 (ab191857) for IHC were purchased from Abcam. D-luciferin (JKG213) was purchased from JINGKEBIO. Goat anti-rabbit IgG (ab150077) was purchased from Abcam; anti-GFAP (16825-1-AP) was purchased from Proteintech; anti-SREBP1 (ab191857) for IHC-P was purchased from Abcam; and DAPI was purchased from Solarbio.

### Cell culture

Informed consent was obtained from the patients of General Hospital, Ningxia Medical University. We mechanically isolated fresh glioma tissue according to the method described by Ho [9] to culture primary glioma cells. The U87 and U251 cell lines were purchased from the Chinese Academy of Sciences and authenticated with short tandem repeat (STR). All cells were cultured in DMEM (Gibco, USA) with 10% fetal bovine serum (Gibco, USA) and 1% penicillin/streptomycin (ScienCell, USA) at 37 °C in a humidified atmosphere of 5% CO2.

### Lentivirus infection

The U87 cell lines were plated in a 6-well plate at 50,000 cells per well and allowed to proliferate until 30% confluence before viral transfection. The cells were transfected with PER2 shRNA lentiviral particles and PER2 overexpression lentiviral particles (Genechem Biotechnology, Shanghai) at a multiplicity of infection (MOI) of 10, replaced with new medium 16 hours after transfection. For determination of the transfection efficacy, the level of PER2 was determined by western blotting after 72 h of transfection.

### Cell viability assay

The cells were plated in 96-well plates at a density of 5000 cells per well with DMEM (10% FBS). After treatment with different concentrations of LbGP, 10 µl of CCK-8 reagent was added to each well for 1 h at 37 °C. Cell viability was detected using a Multimode plate reader (PerkinElmer, US) at 450 nm.

### Migration assay

The cells were plated in 6-well plates, and when the cell density reached 90% confluence, a straight line was drawn with a pipette tip and photographed under a microscope. After treatment with LbGP for 24 h, the cells were observed under a microscope and photographed. Three visual fields were randomly selected in each group for analysis.

### Invasion assay

DMEM and Matrigel were mixed in the upper chamber at a ratio of 8:1 and incubated for 30 min. Then, 10,000 cells were added to the upper chamber of the Transwell inserts with 5% FBS and attracted by 20% FBS in the lower chamber. After treatment with LbGP for 24 h, the cells in the upper chamber were fixed in 4% paraformaldehyde solution for 20 minutes, stained with 0.1% crystal violet staining solution for 15 minutes, and then washed with PBS to remove excess crystal violet. The number of cells that had invaded was counted using a light microscope (Olympus, Japan) at 100× magnification. Three visual fields were randomly selected in each group for analysis.

### Lipid quantitative analysis

Cell samples were analyzed under the following chromatographic and mass spectrometry conditions after a series of pretreatments. The chromatographic conditions were as follows: column temperature: 55 °C; mobile phase A: acetonitrile: water = (60:40, V/V), containing 0.1% FA and 10 mM NH4COOH; mobile phase B: acetonitrile:isopropanol = (10:90, V/V), containing 0.1% FA and 10 mM NH4COOH; flow rate: 0.35 mL/min; and injection volume: 5 µl. The mass spectrometry system used was a Qtrap5500 mass spectrometry detection system from AB Sciex, USA. High-throughput analysis of more than 1000 lipids was performed using schedule-MRM mode. A total of 532 lipids were detected in this lipid-targeted detection. The standard for differential metabolite screening is that the VIP value of the first principal component of the OPLS-DA model is >1, and the *p* value of the *t* test is <0.05. The heat map was performed with R language software. The volcano map was performed with GraphPad Prism software 8.0.A. Luming Biotech Co.,Ltd. (Shanghai, China) provided relevant technical support.

### Flow cytometry for apoptosis analysis

An Annexin V-FITC apoptosis detection kit (BD Biosciences, USA) was used to assess the cells, and flow cytometry (BD Biosciences, USA) was used to detect apoptosis. FlowJo V10 software was used for analysis.

### Immuno-fluorescence

The cells were seeded onto cell slides in a 24-well cell culture plate. After fixation with 4% paraformaldehyde, the cells were permeabilized in 0.1% Triton X-100, blocked for 30 min, and incubated with primary anti-SREBP1 (ab191857, 1:250, Abcam, USA) at 4 °C overnight. Then, the cells were incubated with secondary antibody (ab150077, 1:500, Abcam, USA) for 1 h. The cell nuclei were stained with DAPI and observed with a fluorescence microscope (Olympus, Japan).

### Western blot

The cells were lysed by RIPA buffer, and the supernatant was taken to determine the protein concentration. Proteins with different molecular weights were separated by SDS‒PAGE and transferred to PVDF membranes. The membrane was incubated in 5% milk for 1 h at room temperature and with the primary antibody at 4°C overnight. After TBST washes, the membrane was incubated with secondary antibody for 1 h. All bands were visualized on a gel imaging system (Amersham, USA) using an ECL kit (Seven Biotech, China).

### Immunohistochemistry

All patient specimens were obtained with the informed consent of the patients. Tissue sections were blocked with 3% BSA after antigen retrieval and incubated with anti-SREBP1 (ab191857, 1:250, Abcam, USA) at 4 °C overnight. Then, the samples were incubated with secondary antibody (ab150077, 1:500, Abcam, USA) for 1 h. After PBS washes, DAB staining was performed for 5 minutes, followed by counterstaining with hematoxylin for 3 min. The evaluation criteria for staining grade are as follows: brown (3 points), yellow (2 points), light yellow (1 point), and unstained (0 points). The staining degree evaluation criteria are as follows: the percentage of stained cells ≤ 5% (0 points), 6–25% (1 point), 26–50% (2 points), 51–75% (3 points), and 76–100% (4 points). The score for each sample is the product of the grade score and the staining score. If the score was >2, the sample had strong staining, and if the score was ≤2, the sample had weak staining, which represent high and low expression respectively. Images were obtained from the tissue sections and analyzed using a microscope (Olympus, Japan).

### Real-time quantitative PCR

According to the instructions, total RNA was extracted with TRIzol reagent (Invitrogen, USA), RNA was reverse transcribed into cDNA by HiScript ®III SuperMix for qPCR (Vazyme, China), and ChamQ Universal SYBR qPCR Master Mix (Vazyme, China) and the IQ5 PCR System were used (Bio-Rad, USA) to analyze the relative expression of mRNA. The primer sequences used were as follows: Per2 forward, 5’-TTGGACAGCGTCATCAGGTA-3’ and reverse, 5’-TCCGCTTATCACTGGACCTT-3’; SREBP1 forward, 5’-GACAGCCCAGTCTTTGAGGA-3’ and reverse, 5’-CAGGACAGGCAGAGGAAGAC-3’; FASN forward, 5’-CACAGGGACAACCTGGAGTT-3’ and reverse, 5’-ACTCCACAGGTGGGAACAAG-3’; SCD forward, 5’-CGACGTGGCTTTTTCTTCTC-3’ and reverse, 5’-CCTTCTCTTTGACAGCTGGG-3’; ACACA forward, 5’-AGTGGGTCACCCCATTGTT-3’ and reverse, 5’TTCTAACAGGAGCTGGAGCC-3’; Bmal1 forward, 5’-CTCCTCCAATGTGGGCATCAA-3’, and reverse, 5’-GGTGGCACCTCTTAATGTTTTCA-3’; CLOCK forward, 5’-TGCGAGGAACAATAGACCCAA-3’ and reverse, 5’-ATGGCCTATGTGTGCGTTGTA-3’; Cry1 forward, 5’-CTCCTCCAATGTGGGCATCAA-3’ and reverse, 5’-CCACGAATCACAAACAGACGG-3’; Cry2 forward, 5’-TCCCAAGGCTGTTCAAGGAAT-3’ and reverse, 5’-TGCATCCCGTTCTTTCCCAAA-3’; PER1 forward, 5’-AGTCCGTCTTCTGCCGTATCA-3’ and reverse, 5’-AGCTTCGTAACCCGAATGGAT-3’; PER3 forward, 5’-GCAGAGGAAATTGGCGGACA-3’ and reverse, 5’-GGTTTATTGCGTCTCTCCGAG-3’.

### Glioma xenograft mouse model

Before the model was established, shNC U87 and shPer2 cells were first transducted with lentivirus to stably express luciferin. 6-week-old male BALB/c nude mice were purchased from Charles River Company (Beijing, China), 2×10^7^/ml cell suspension was prepared for injection. After isoflurane anesthesia, the mouse head was fixed with the aid of a stereotaxic instrument. After we confirmed that the anesthetic effect was satisfactory, the scalp was vertically cut in the middle of the mouse head, and a drill was used to drill 2 mm to the right of the midpoint of the anterior and posterior fontanelles. A circular hole with a diameter of 2 mm was used to slowly inject 5 μl of the cell suspension at a depth of 2.5 mm. After the injection was completed, the needle was slowly pulled out, and the scalp was sealed with glue. An in vivo imaging system (IVIS) was used. D-Luciferin (JKG213) was intraperitoneally injected before isoflurane anesthesia, and imaging was performed in the in vivo imager 10 min later, (*n* = 4 per group). We used the second batch of mice alone to study the survival of mice (*n* = 5 per group). The investigator was blinded to the group allocation and result analysis during the experiment. All experimental procedures were approved by the Animal Center of Ningxia Medical University.

### Statistical analysis

A *t test* was used to for each two-group comparison. *One-way* analysis of variance (ANOVA) was used for multiple comparisons. Correlation analysis between two variables using *chi-square* analysis. Kaplan-Meier survival analysis was used to compare the survival rates of patients in different groups. All experiments were performed at least three times. *P* < 0.05 was considered significant.

## Results

### LbGP inhibits proliferation, migration and invasion, and promotes apoptosis of glioma cells

**Fig. 1:**
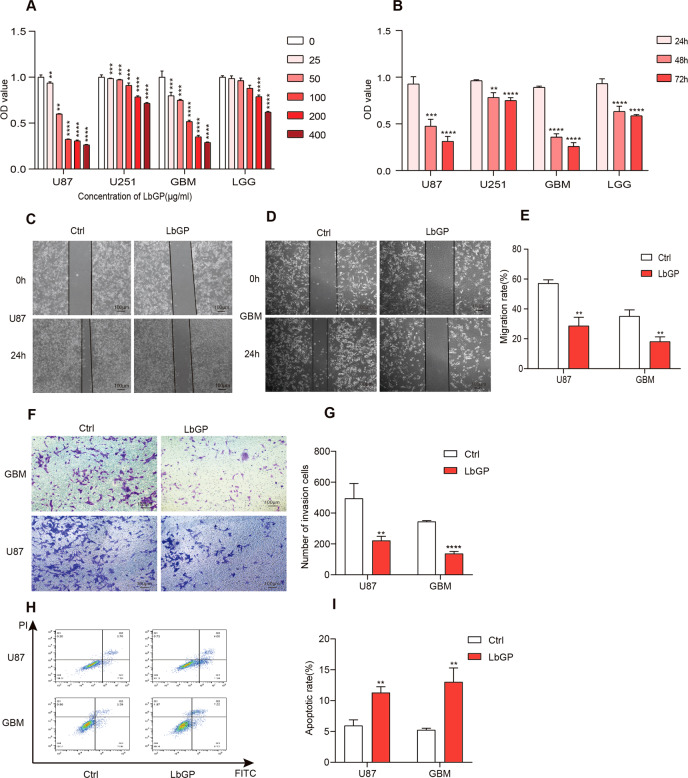
LbGP inhibits proliferation, migration and invasion, and promotes apoptosis of glioma cells. **A** Four kinds of glioma cells (U87, U251, GBM, LGG) were treated with LbGP at different concentrations, and cell viability was determined by CCK-8 assays. **B** Four kinds of glioma cells (U87, U251, GBM, LGG) were treated with 200 µg/ml LbGP for 24 h,48 h and 72 h, and cell viability was determined by CCK-8 assays. **C**–**E** Migration assays of the and GBM and U87 cells treated with 200 µg/ml LbGP for 24 h. **F**–**G** Transwell invasion assays of the GBM and U87 cells treated with 200 µg/ml LbGP for 24 h. **H**–**I** The apoptosis rates of the U87 cells and GBM cells treated with 200 µg/ml LbGP for 48 h were determined by flow cytometry. The data are presented as the mean ± SD of three replicates. *P < 0.05, **P < 0.01, ***P < 0.001, ****P < 0.0001.

LbGP can inhibit the viability of hepatoma cells (SMMC-7721 and HepG2), cervical cancer cells (HeLa), gastric carcinoma cells (SGC-7901), and human breast cancer cells (MCF-7) [14]. To study the effect of LbGP on the viability of glioma cells, we used a CCK-8 assay to test the viability of glioma cells with different concentrations of LbGP (Tianren Bio-engineering Co., Ltd., Zhongning, China) (Fig. 1A). To ensure that the tested cells had the characteristics and properties of primitive cells, we isolated and cultured primary glioma cells from two patients. The pathological results of the two patients indicated that they had glioblastoma (GBM) and WHO grade II (LGG), and cells were identified by GFAP immunofluorescence (Fig. SA). When the LbGP concentration was 200 µg/ml, the cell viability was significantly affected. According to our results, GBM and U87 cells are sensitive to LbGP (Fig. 1B). LbGP at a concentration of 200 µg/ml inhibited the migration and invasion of U87 and GBM cells (Fig. 1C–G). Apoptosis of U87 cells and primary GBM cells was determined by flow cytometry (Fig. 1H–I).

### LbGP upregulates the expression of PER2 through the PKA-CREB-PER2 pathway

**Fig. 2:**
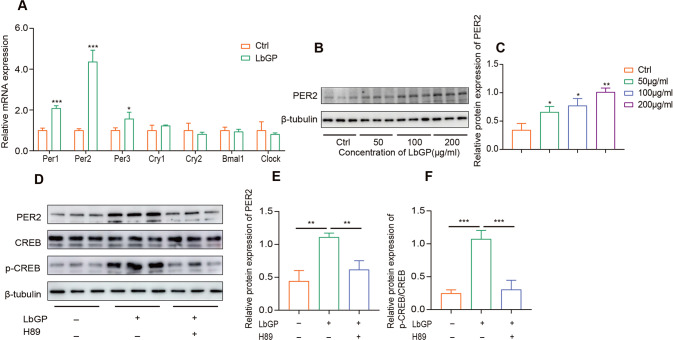
LbGP upregulates the expression of PER2 through the PKA-CREB-PER2 pathway. **A** RT-qPCR was performed to detected 7 clock genes in U87 cells treated with 200 µg/ml of LbGP for 6 h. **B**, **C** Protein expression levels of PER2 in the control and LbGP treated U87 cells for 6 h. **D**–**F** U87 cells were treated with LbGP for 6 h, H89 concentration was at 30 µM. The expression of protein was measured by western blotting. The data are represented as the mean ± SD of three replicates. *P < 0.05, **P < 0.01, ***P < 0.001.

The effectiveness of numerous drugs has been confirmed to be associated with the circadian rhythm of biochemical processes under the control of circadian clock genes, and LBP is involved in the regulation of biorhythm genes [15]. To determine the effect of LbGP on circadian genes, we performed RT‒qPCR on 7 biological rhythm genes to verify whether LbGP changed the expression of these genes. The results suggested that LbGP upregulated the expression of Per1, Per2 and Per3, and Per2 was significantly upregulated (Fig. 2A). Further western blot results showed that LbGP promoted protein expression in a dose-dependent manner (Fig. 2B, C). Our results suggest that the upregulation of PER2 by LbGP is accompanied by the upregulation of p-CREB, and neither p-CREB nor PER2 can be upregulated when this process is inhibited by a PKA inhibitor (H89) (Fig. 2D–F). Based on the above results, we infer that PER2 is the target gene of LbGP and that LbGP promotes the expression of PER2 by activating the PKA-CREB pathway.

### LbGP reduces tumor growth depending on the expression of PER2

**Fig. 3:**
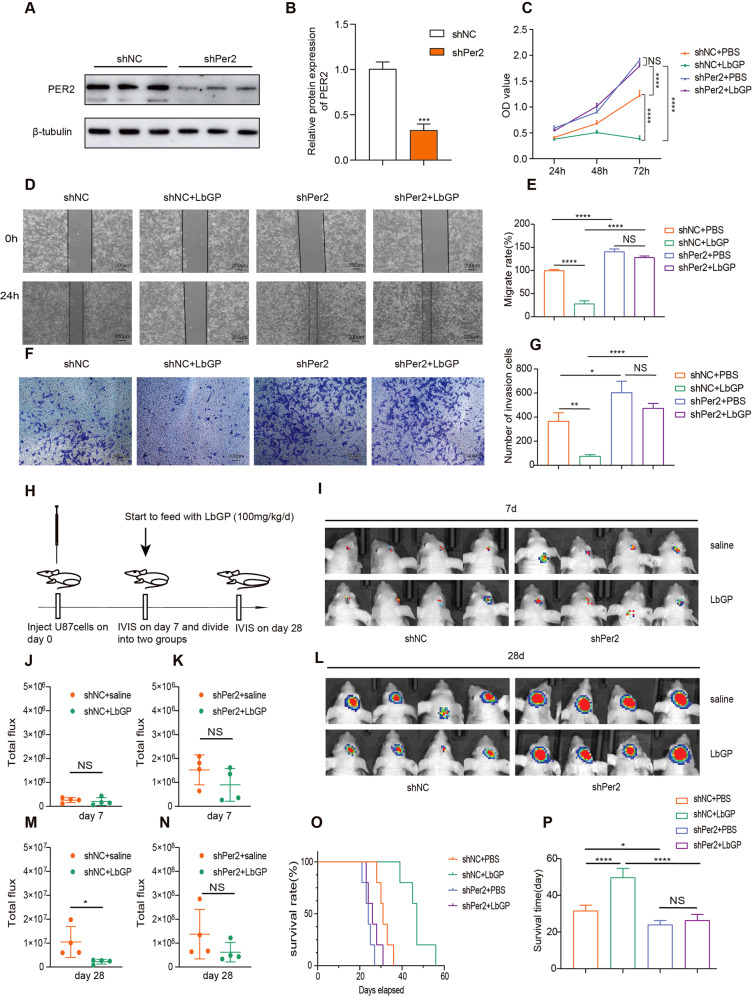
LbGP reduces tumor growth depending on the expression of PER2. **A, B** The expression of PER2 was measured by western blotting after silenced by RNA lentivirus. **C** shNC or shPer2 U87 Cells were treated with LbGP or PBS for 24 h, and the cell viability was determined by CCK-8 assay. **D**, **E** Migration assays of shNC or shPer2 U87 cells treated with 200 µg/ml of LbGP for 24 h. **F, G** Transwell invision assays of shNC or shPer2 U87 cells treated with 200 µg/ml of LbGP for 24 h. **H** Schedule of inject cells、feed LbGP and IVIS. **I**–**K** IVIS image and total flux results on day 7. **L**–**N** IVIS image and total flux results on day 28 of different groups. **O, P** Survival rates and time of different groups. The data are represented as the mean ± SD of three replicates. *P < 0.05, **P < 0.01, ***P < 0.001, ****P < 0.0001.

Per2 can inhibit the proliferation of a variety of cancer cells [7]. To study the role of PER2 in the antitumor effect of LbGP, we knocked down PER2 and obtained shNC and shPer2 U87 cell lines (Fig. 3A, B). First, we tested the cell viability by CCK-8 assays. The results showed that LbGP could still reduce the proliferation of shNC U87 cells; however, we could barely detect an effect of LbGP on cell viability in the Per2-silenced cells. At the same time, U87 cell proliferation increased when PER2 was silenced (Fig. 3C). Similarly, silencing Per2 blocked the ability of LbGP to inhibit cell migration and invasion (Fig. 3D–G). In addition, we further tested the performance of LbGP in vivo. We established an animal model of intracranial tumorigenesis, and shNC U87 cells and shPer2 U87 cells were transplanted into the brains of the mice on day 0 (Fig. 3H). On day 7, we first (IVIS) confirmed the success of tumor growth in the brain, and the mice transplanted with the shNC U87 cells and the shPer2 U87 cells were randomly divided into two groups fed LbGP or the same volume of saline (Fig. 3I–K). IVIS on the 28th day showed that LbGP inhibited the in vivo growth of the shNC U87 cells but not the shPer2 cells (Fig. 3L–N). In the survival analysis of the mice, the group fed LbGP showed a longer survival time, but there was no significant difference in survival time between the mice transplanted with the shPer2 U87 cells fed LbGP and the mice without LbGP (Fig. 3O, P). These results indicate that the in vitro and in vivo tumor-suppressive effects of LbGP depend on the expression of PER2.

### LbGP reduces the de novo lipid synthesis and metabolism of glioma cells depending on the expression of PER2

**Fig. 4:**
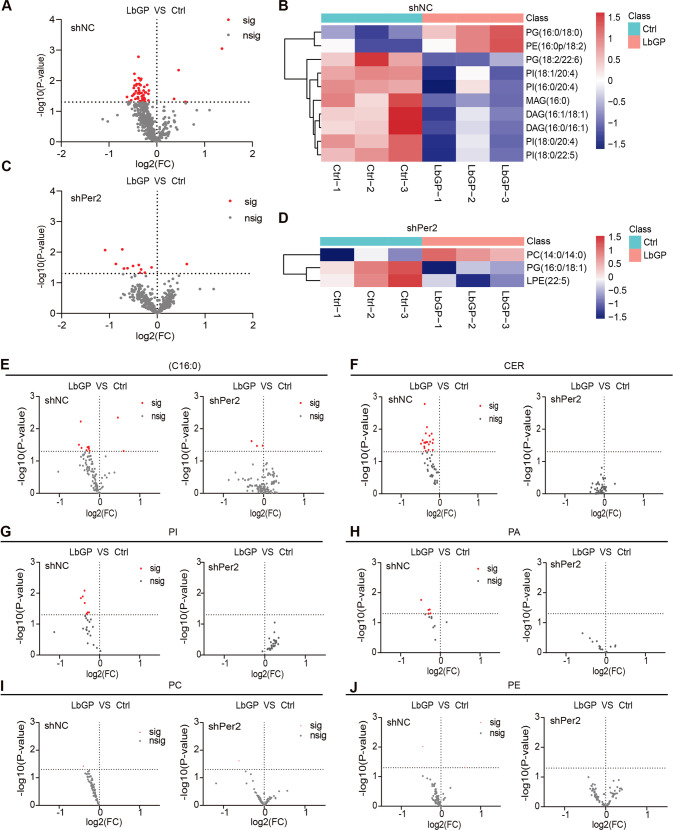
LbGP reduces the de novo lipid synthesis and metabolism of glioma cells depending on the expression of PER2. **A** Volcano plots showing fold changes in lipids in the shNC U87 cells treated with 200 µg/ml LbGP for 24 h. **B** Heatmap showing the specific lipids in the shNC U87 cells treated with 200 µg/ml LbGP for 24 h (VIPå 1). **C** Volcano plots showing fold changes in lipids in the shPer2 U87 cells treated with 200 µg/ml LbGP for 24 h. **D** Heatmap showing the specific lipids in the shNC U87 cells treated with 200 µg/ml LbGP for 24 h (VIPå 1). **E**–**J** Volcano plots showing fold changes in the lipids (16:0), CER, PA, PI, PC, and PE in the shNC or shPer2 U87 cells. The data are represented as the mean ± SD of three replicates. Significance is represented as *P < 0.05. Not significant is represented as *P ≥ 0.05. OPLS-DA was used to analyze differentially abundant metabolites. Variable importance in projection (VIP) was used to measure the impact strength and explanatory power of the expression patterns of each metabolite on the classification and discrimination of each group of samples.

Since LbGP altered the changes in circadian clock genes in the above results, the biological clock gene is closely related to metabolism, and LbGP can reduce serum triglycerides [16]. Therefore, we performed a LC/MS pseudotargeted lipidomic analysis to monitor the effect of LbGP on lipid metabolism in the shNC or shPer2 U87 cells. The results showed that LbGP significantly reduced the levels of various lipids in the shNC U87 cells (Fig. 4A, B). However, LbGP decreased the levels of few kinds of lipids in the shPer2 U87 cells (Fig. 4C, D). Phospholipids are the main component of cell membranes. LbGP significantly decreased multiple kinds of phospholipids, such as CER, PI and PA. However, LbGP did not significantly change the content of the above substances in the Per2-silenced cells (Fig. 4E–J).

### LbGP inhibits lipid synthesis by downregulating SREBP1c and its target genes depend on the expression of PER2

**Fig. 5:**
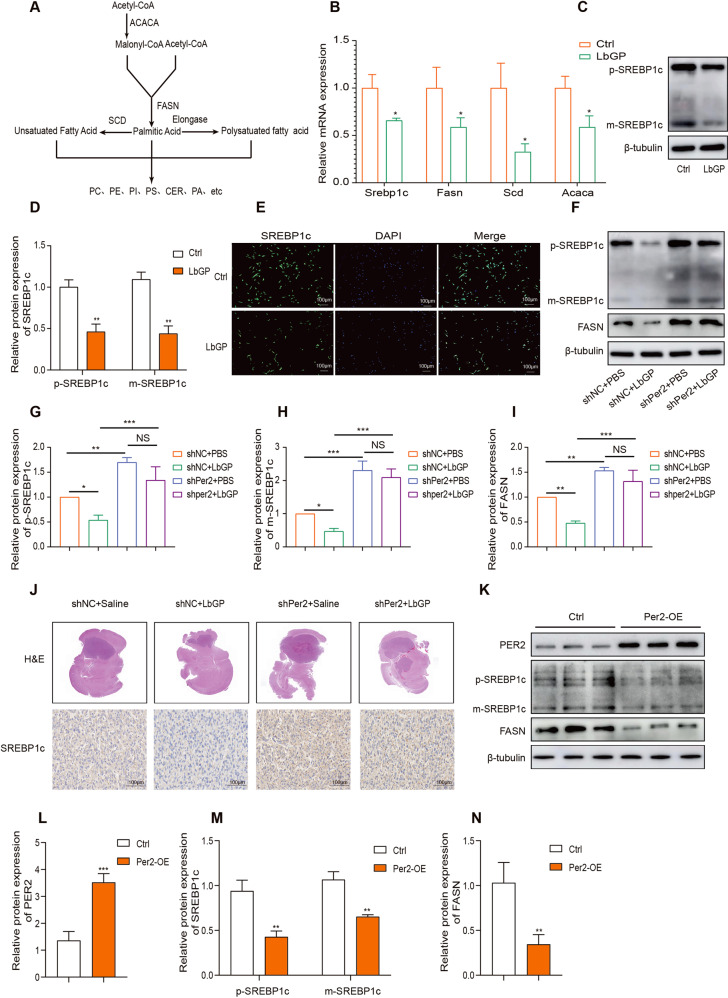
LbGP inhibits lipid synthesis by downregulating SREBP1c and its target genes depend on the expression of PER2. **A** Process of lipid synthesis and metabolism. **B** RT‒qPCR was performed to detect 4 lipid-related genes in the U87 cells treated with 200 µg/ml LbGP for 6 h. **C, D** Western bolt of the U87 cells treated with 200 µg/ml LbGP for 6 h. E ICF of the U87 cells treated with 200 µg/ml LbGP for 6 h. **F**–**I** Western blot was performed to assess protein expression of the shNC or shPer2 U87 cells treated with 200 µg/ml LbGP or PBS for 6 h. **J** The expression levels of SREBP1c were examined by immunohistochemistry in animal specimens. **K**–**N** The protein expression of SREBP1c, FASN and PER2 was measured by western blotting. The data are represented as the mean ± SD of three replicates. *P < 0.05, **P < 0.01, ***P < 0.001.

The above results suggest that LbGP reduces the synthesis of various lipids and that SREBP1c and its target genes, such as ACACA, FASN, and SCD, regulate the process of lipid synthesis (Fig. 5A). SREBP1c plays a critical role in the cell growth of glioma. Based on the LC/MS results, we wondered whether LbGP affected the expression of SREBP1c and its target genes. RT‒PCR were performed and indicated that LbGP reduces the mRNA level of SREBP1 and its target genes FASN, SCD and ACACA (Fig. 5B). Immunofluorescence and western blot showed that LbGP decreased the expression of SREBP1 (Fig. 5C–E). Further western blot experiments suggested that when Per2 was silenced, both the precursor form (p-SREBP1c) and mature form of SREBP1c (m-SREBP1c) were upregulated, and LbGP could not downregulate SREBP1c (Fig. 5F–I). Furthermore, above results suggest that SREBP1c expression is negatively regulated by PER2. We found a similar result in the SREBP1c immunohistochemical analysis of animal specimens (Fig. 5J). To test this hypothesis, we overexpressed Per2 and found that Srebp1c was downregulated (Fig. 5K–N).

### Correlations between PER2 and SREBP1c in clinical samples from patients

**Fig. 6:**
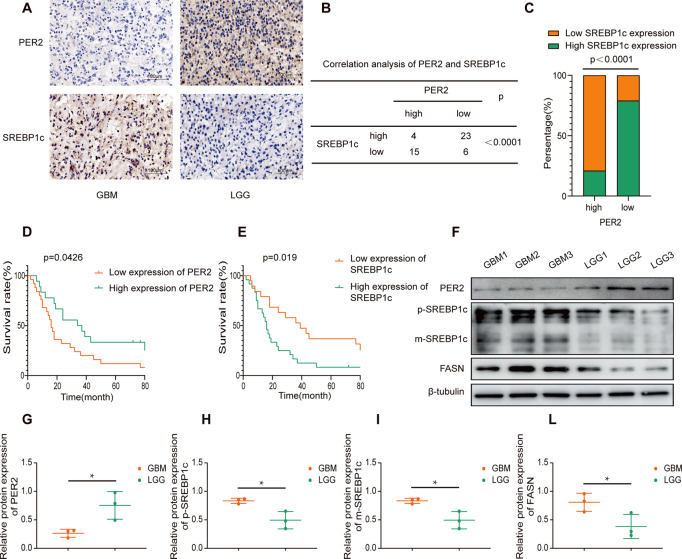
Correlations between PER2 and SREBP1c in clinical samples from patients. **A** The expression levels of PER2 and SREBP1c were examined by immunohistochemistry in glioma tissue of different grades. **B, C** PER2 and SREBP1c immunohistochemistry score of high or low in glioma tissue from 48 patients. The correlation was assessed with *χ2* test. **P < 0.01. **D, E** Patients’ Survival rates with high expression of PER2 or SREBP1c. **F**–**L** Protein expression of FASN、SREBP1c and PER2 in primary glioma cells from 6 patients.

The above results suggest that PER2 has a negative regulatory relationship with SREBP1c. To further verify this result, we collected 48 surgical specimens from glioma patients at the General Hospital of Ningxia Medical University from January 2014 to December 2016, performed SREBP1c and PER2 immunohistochemical staining, and scored the results of immunohistochemical staining according to the two grades. Our immunohistochemical results showed that there was a negative correlation between PER2 expression and SREBP1c expression (Fig. 6A–C). We followed them up by telephone, and 43 patients got the results of the follow-up. According to the expression levels of PER2 and SREBP1c, we obtained their survival curves. The results suggest that the patient group with high expression of PER2 and low expression of SREBP1c has a longer survival time (Fig. D-E). We cultured 6 patient-derived primary glioma cells; 3 of the patients were diagnosed with glioblastoma (WHO IV) and 3 of them had low-grade glioma (WHO II). Western blot results showed that the glioblastoma samples had high FASN and SREBP1c expression and low PER2 expression (Fig. F-L).

### PER2 negatively regulates SREBP1c by inhibiting the PI3K/AKT/mTOR pathway

**Fig. 7:**
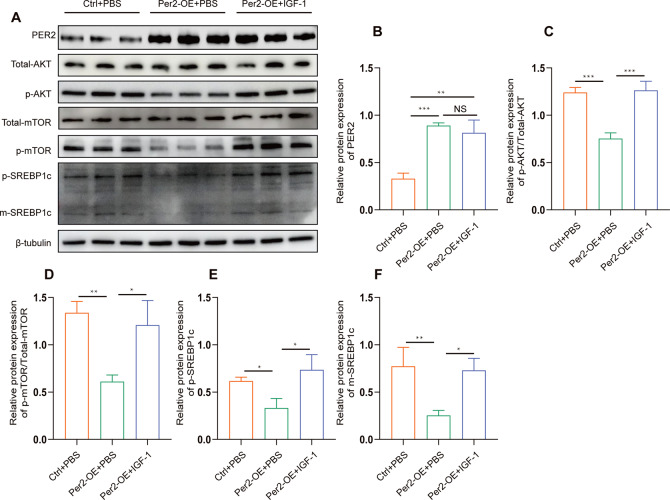
PER2 negatively regulates SREBP1c by inhibiting the PI3K/AKT/mTOR pathway. **A**–**F** Overexpression of Per2 inhibited the expression of SREBP1c via downregulation of the PI3K/AKT/mTOR signaling pathway. Expression levels of PER2, AKT, p-AKT, mTOR, p-mTOR, SREBP1c, was detected with Western blot. The data are represented as the mean ± SD of three replicates. *P < 0.05, **P < 0.01, ***P < 0.001, ****P < 0.0001.

PER2 inhibits the proliferation of oral squamous cell carcinoma and promotes the apoptosis of OSCC cells, which is dependent on the PI3K/AKT/mTOR signaling pathway [17]. PI3K/AKT pathway promotes fatty acid anabolism [18]. We speculate that the regulation of SREBP1c by PER2 may be achieved through the inhibition of PI3K/AKT/mTOR pathway.The western blots were used to verify our speculation. The results suggested that when PER2 is overexpressed, the PI3K/AKT/mTOR pathway is inhibited, overexpression of Per2 inhibits the PI3K/AKT/mTOR pathway. When we use the PI3K/AKT pathway activator IGF-1, PER2 lost its inhibition of SREBP1c (Fig. 7A–F). These results suggest that the negative regulation of SREBP1c by PER2 may be achieved through the inhibition of PI3K/AKT/mTOR pathway.

## Discussion

Previous studies have shown that LbGP prevents depression by inhibiting abnormal neuronal activity and microglial activation in the lateral habenular nucleus [19]. In addition, LbGP has significant antioxidant and immune regulatory effects. In terms of antitumor effects, LBGP inhibits tumor cell growth through cell cycle arrest and apoptosis [14]. In this study, four kinds of glioma cells were used to confirm that LbGP could inhibit the viability of glioma cells. Further experimental results showed that LbGP could inhibit the proliferation, migration and invasion of glioma cells but also induce apoptosis of glioma cells.

Clock genes have been reported to be associated with a variety of cancers [20–22]. Mice lacking the PER2 gene are susceptible to cancer and show suppressed tumor growth [23]. Early studies by other members of our team have shown that the clock gene PER2 can inhibit the proliferation of tumor cells [7]. At present, it is not clear how clock genes affect the biological function of tumors. This study confirmed that LbGP can inhibit the proliferation of glioma cells and that LbGP upregulates the expression of PER2 in the PKA-CREB pathway. In addition, this inhibitory effect of LbGP on cells was controlled by PER2. These results indicated that PER2 plays a crucial role in the process.

The proliferation of cancer cells depends on environmental lipids when electron acceptors are limited [24]. Lipids are the main components of cell membranes, and are also important for energy storage and transport. Lipids can also act as intracellular and intercellular signaling molecules that have extensive effects on cell physiology [13, 22]. The synthesis of lipids affects tumor cell proliferation, migration, invasion and apoptosis [14, 21]. Clock genes control the transcription of a series of downstream genes in a circadian rhythm [10]. The correlation between lipid metabolism and clock genes is still unclear. In this study, LC/MS metabonomics was used to show that LbGP can inhibit the synthesis of various lipids in U87 cells, and LbGP downregulated the expression of SREBP1c and its downstream genes. Although SREBP-1 was discovered more than 20 years ago [25], the development of clinically feasible SREBP-1 inhibitors has not been successful. Our study found for the first time that LbGP can be used as an SREBP1c inhibitor to inhibit lipid synthesis in glioma cells. Our further results showed that the regulation of SREBP1c expression is controlled by PER2, and these results indicate that PER2 plays a crucial role in the process of lipid inhibition by LbGP.

Hundreds of lipid biosynthesis and fatty acid oxidation genes in peripheral tissue are rhythmically activated and inhibited by biological clock genes, which also provides a direct mechanism for lipid regulation [12]. Hundreds of lipids in mammalian peripheral tissue show circadian rhythmicity. The source of their rhythmicity is the expression rhythm of SREBP1c and its downstream genes [26]. At present, the relationship between clock genes and lipid metabolism is still unclear. Our research shows that there is a negative correlation between the expression of PER2 and SREBP1c. moreover, PER2 downregulates SREBP1c possibly by inhibiting the PI3K/AKT/MTOR pathway. In conclusion, Our results suggest that LbGP can be used as an alternative drug to inhibit glioma proliferation, we speculate that LbGP suppresses the expression of SREBP1c by up-regulating Per2, thus inhibiting lipid synthesis and metabolism in gliomas.

## Supplementary information

Original data for western blots

Original data for qPCR

## Data Availability

The specific data and information support the findings of this study are available from the corresponding author.
