# Correction to: *Lycium barbarum* glycopeptide targets PER2 to inhibit lipogenesis in glioblastoma by downregulating SREBP1c

**DOI:** 10.1038/s41417-023-00620-3

**Published:** 2023-05-03

**Authors:** Jian Yao, Jian-wen Hui, Yan-jun Chen, Dong-yang Luo, Jiang-shu Yan, Yi-fan Zhang, Yuan-xiang Lan, Xiu-rui Yan, Zhi-hua Wang, Heng Fan, He-chun Xia

**Affiliations:** 1 Ningxia Medical University, Yinchuan, Ningxia Hui Autonomous Region 750004 P.R. China; 2 Department of Neurosurgery, General Hospital of Ningxia Medical University, Yinchuan, Ningxia Hui Autonomous Region 750004 P.R. China; 3 Ningxia Key Laboratory of Stem Cell and Regenerative Medicine, Institute of Medical Sciences, General Hospital of Ningxia Medical University, Yinchuan, Ningxia, Autonomous Region 750004 China

**Keywords:** Biomarkers, Cancer metabolism

Correction to: *Cancer Gene Therapy* 10.1038/s41417-023-00611-4, published online 17 April 2023

In this article, the author name He-chun Xia was incorrectly written as Xe-chun Xia.

The original article has been corrected.

