# Supplementary figures and images for: Lycium barbarum glycopeptide targets PER2 to inhibit lipogenesis in glioblastoma by downregulating SREBP1c
Source: Cancer Gene Ther. 2023 Apr 17;30(8):1084–93. doi: 10.1038/s41417-023-00611-4 (PMC10425286; doi:10.1038/s41417-023-00611-4)

Fig 2B


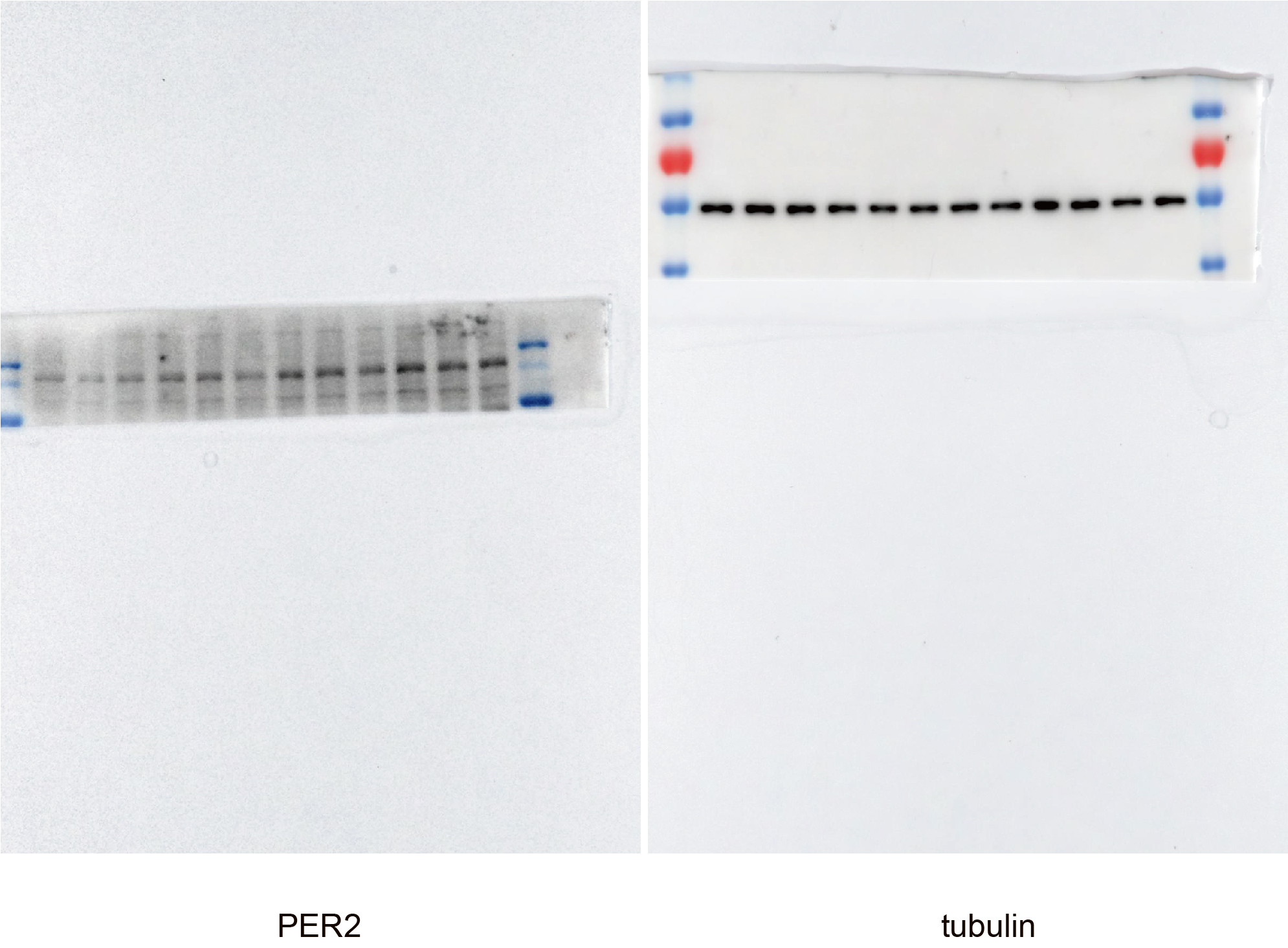


Fig 2D


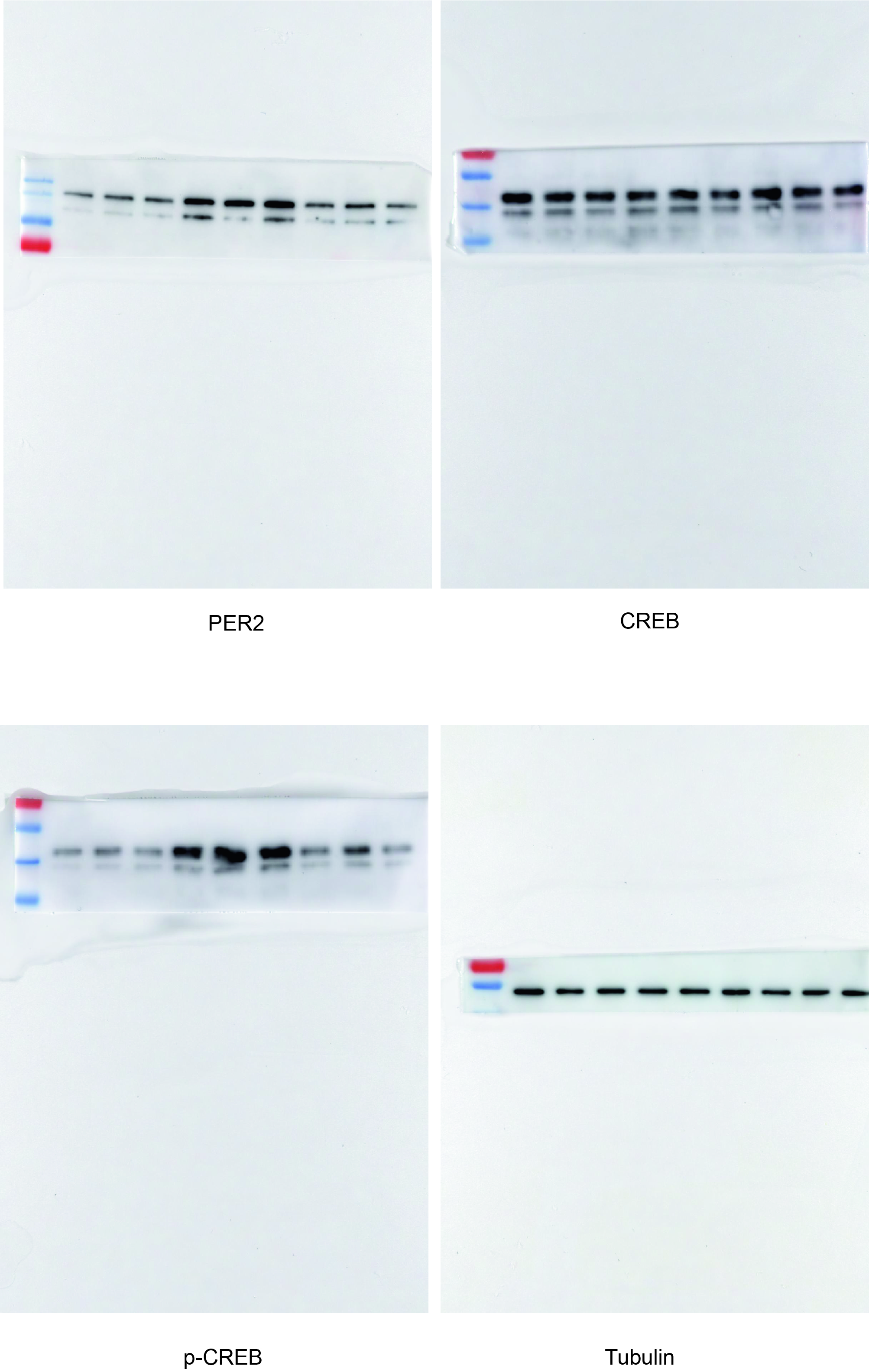


Fig 3A
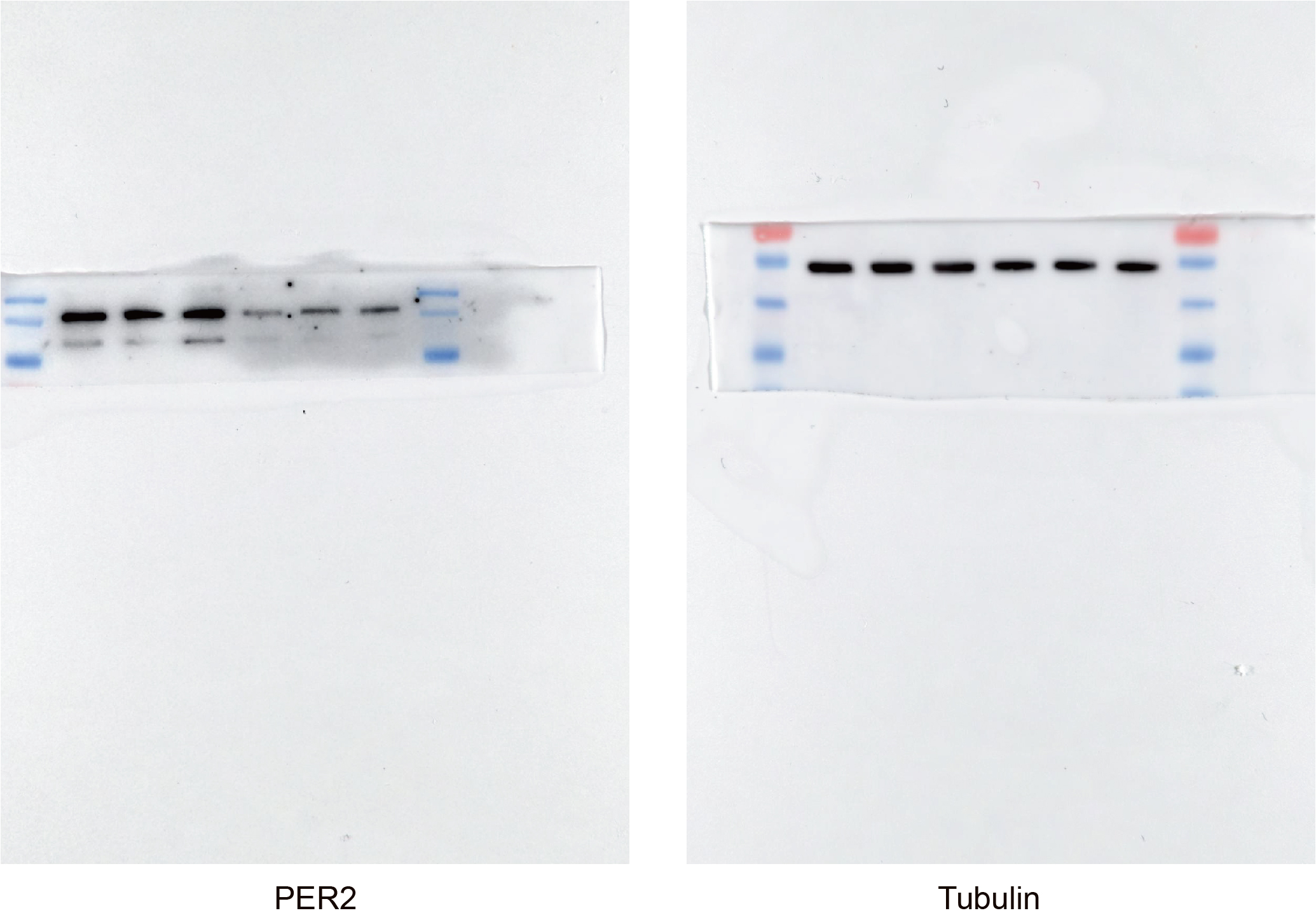


Fig 5C


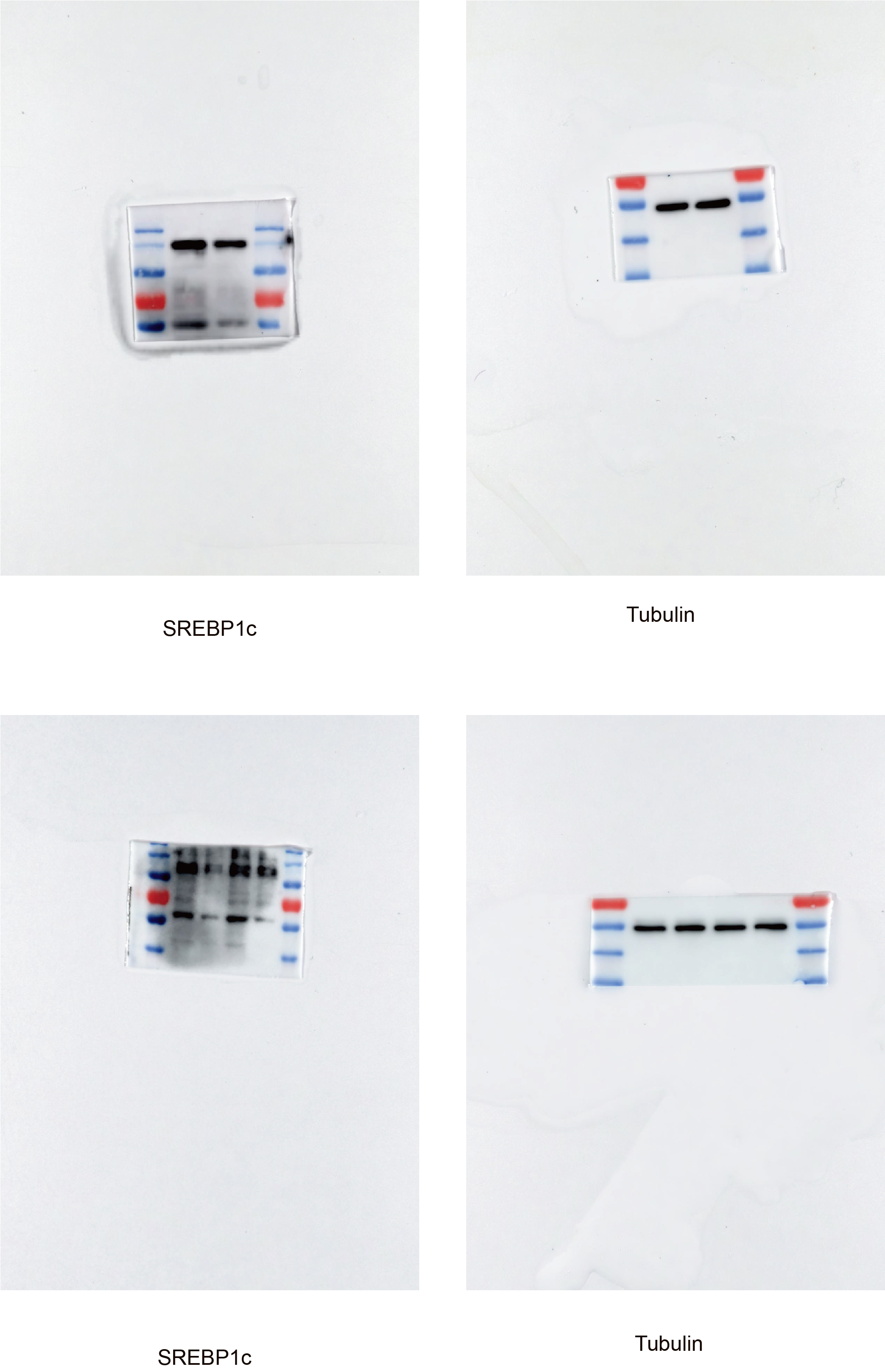


Fig 5F


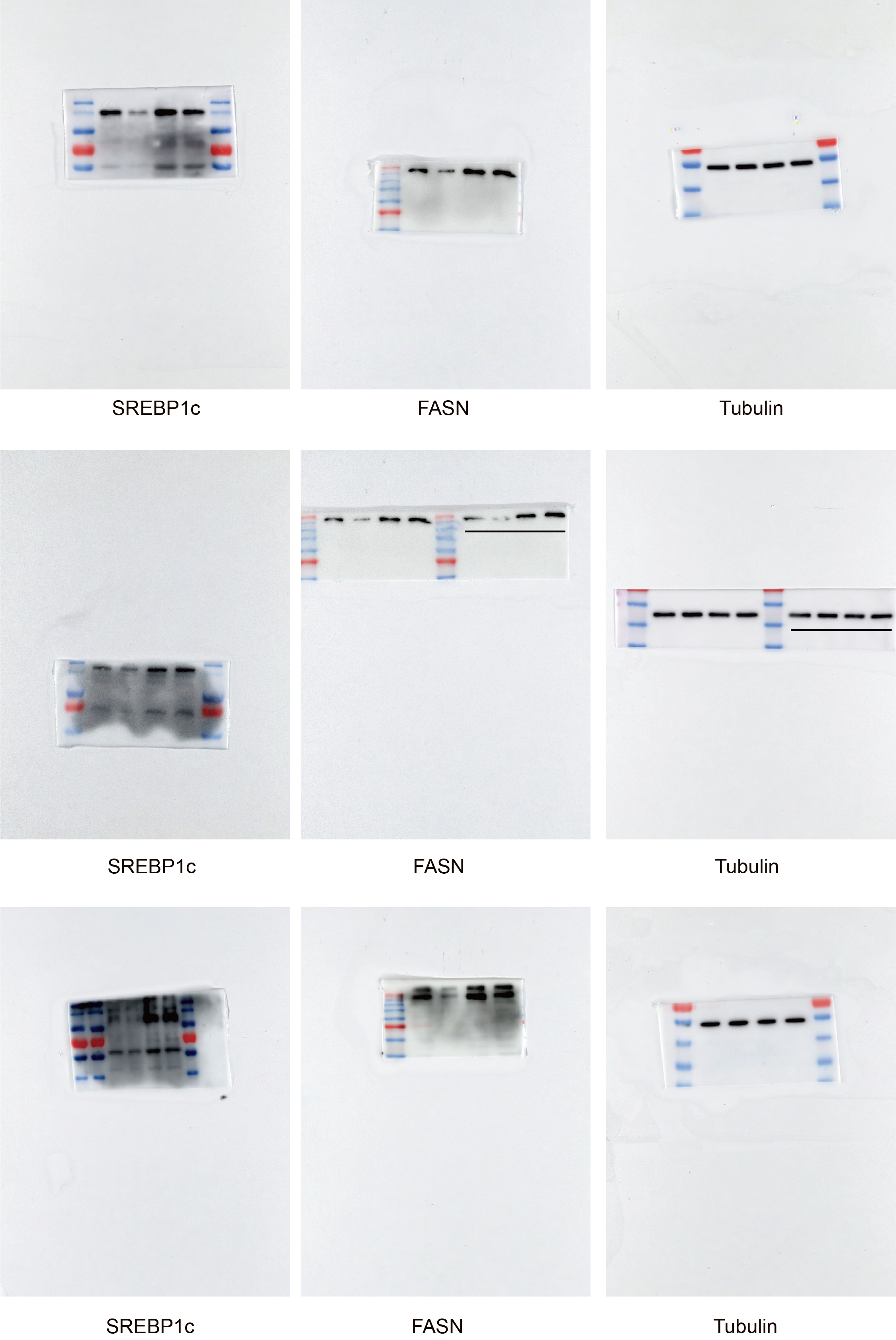


Fig 5K


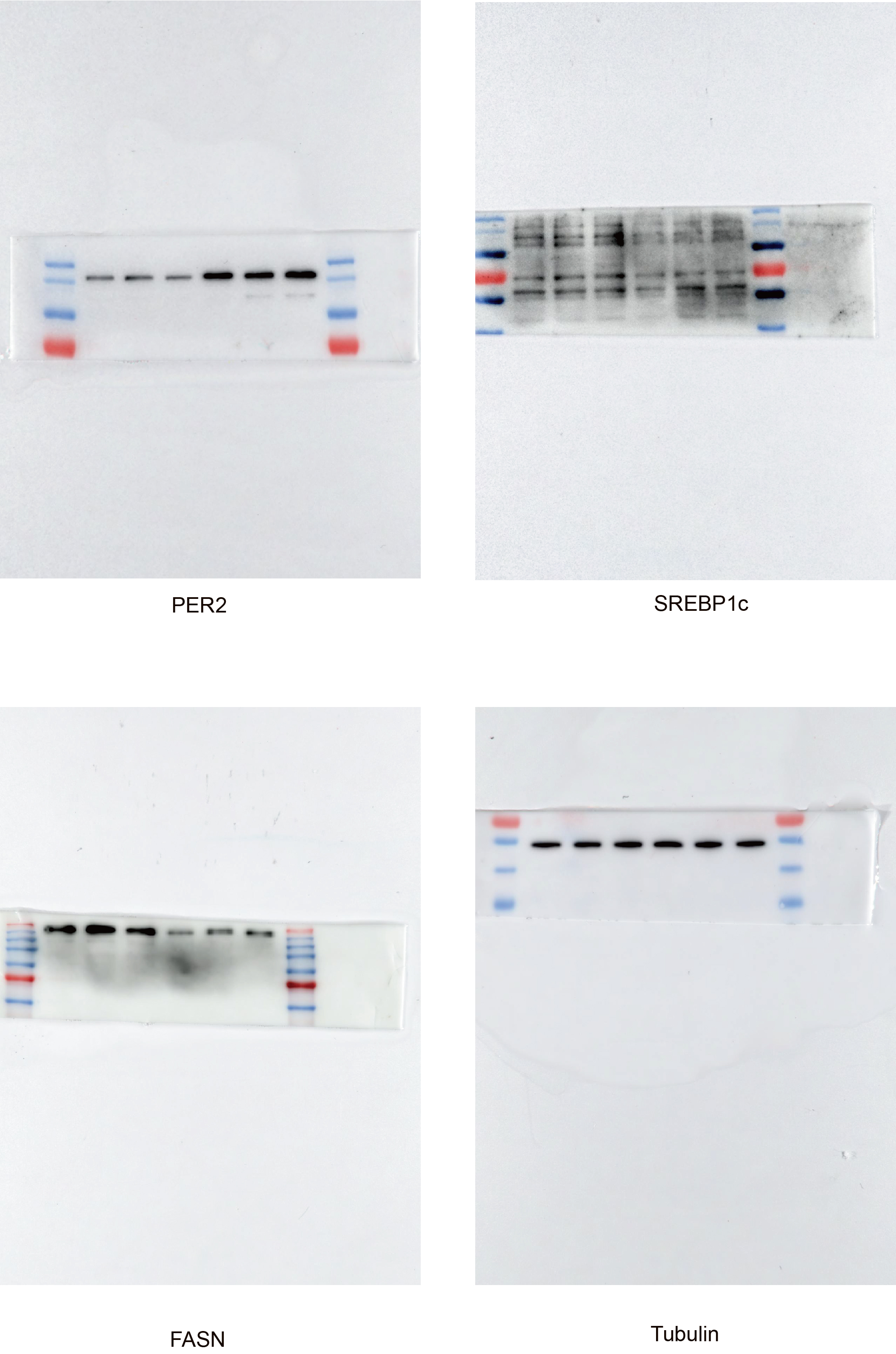


Fig 6A


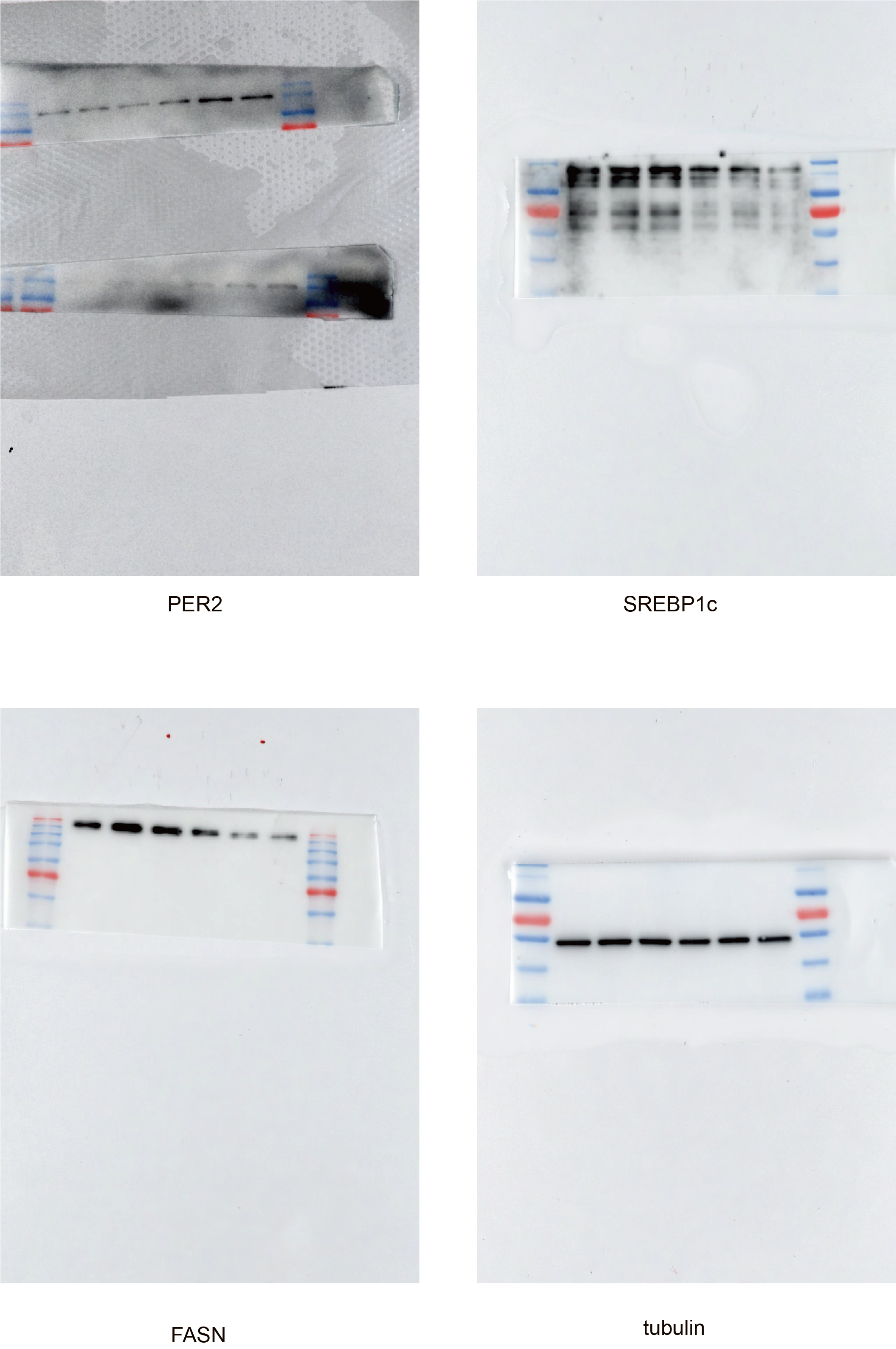


Fig 7A


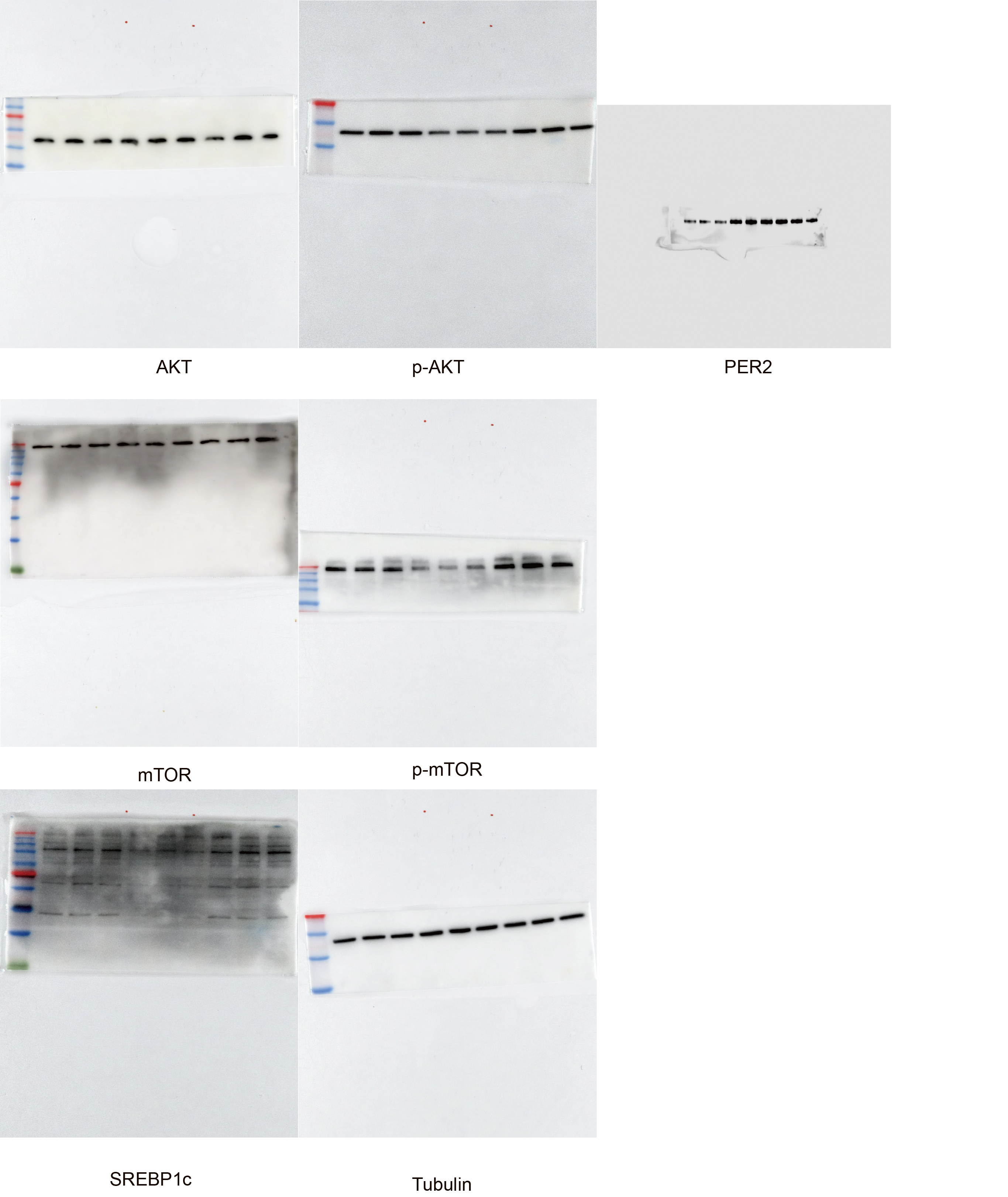

Supplement: Supplementary file 1 — Original data for western blots [file 41417_2023_611_MOESM1_ESM.doc]
